# Current Smoking is Associated with Decreased Expression of miR-335-5p in Parenchymal Lung Fibroblasts

**DOI:** 10.3390/ijms20205176

**Published:** 2019-10-18

**Authors:** Jennie Ong, Anke van den Berg, Alen Faiz, Ilse M Boudewijn, Wim Timens, Cornelis J Vermeulen, Brian G Oliver, Klaas Kok, Martijn M Terpstra, Maarten van den Berge, Corry-Anke Brandsma, Joost Kluiver

**Affiliations:** 1 Department of Pathology and Medical Biology, University Medical Center Groningen, University of Groningen, 9713 GZ Groningen, The Netherlands; j.ong@umcg.nl (J.O.);; 2 Groningen Research Institute for Asthma and COPD (GRIAC), University Medical Center Groningen, University of Groningen, 9713 GZ Groningen, The Netherlands; 3 Department of Pulmonary Diseases, University Medical Center Groningen, University of Groningen, 9713 GZ Groningen, The Netherlands; 4 Respiratory Bioinformatics and Molecular Biology (RBMB) Faculty of Science, University of Technology Sydney, Ultimo, NSW 2007, Australia; 5 Woolcock Institute of Medical Research, Respiratory Cellular and Molecular Biology, The University of Sydney, New South Wales 2037, Australia; 6 School of Life Sciences, University of Technology Sydney, Sydney, New South Wales 2007, Australia; 7 Department of Genetics, University Medical Center Groningen, University of Groningen, 9713 GZ Groningen, The Netherlands

**Keywords:** miRNAs, lung fibroblasts, smoking status, regional methylation

## Abstract

Cigarette smoking causes lung inflammation and tissue damage. Lung fibroblasts play a major role in tissue repair. Previous studies have reported smoking-associated changes in fibroblast responses and methylation patterns. Our aim was to identify the effect of current smoking on miRNA expression in primary lung fibroblasts. Small RNA sequencing was performed on lung fibroblasts from nine current and six ex-smokers with normal lung function. MiR-335-5p and miR-335-3p were significantly downregulated in lung fibroblasts from current compared to ex-smokers (false discovery rate (FDR) <0.05). Differential miR-335-5p expression was validated with RT-qPCR (*p*-value = 0.01). The results were validated in lung tissue from current and ex-smokers and in bronchial biopsies from non-diseased smokers and never-smokers (*p*-value <0.05). The methylation pattern of the miR-335 host gene, determined by methylation-specific qPCR, did not differ between current and ex-smokers. To obtain insights into the genes regulated by miR-335-5p in fibroblasts, we overlapped all proven miR-335-5p targets with our previously published miRNA targetome data in lung fibroblasts. This revealed *Rb1*, *CARF*, and *SGK3* as likely targets of miR-335-5p in lung fibroblasts. Our study indicates that miR-335-5p downregulation due to current smoking may affect its function in lung fibroblasts by targeting *Rb1*, *CARF* and *SGK3*.

## 1. Introduction

Cigarette smoke consists of a complex mixture of thousands of toxic chemicals and over 10^15^ reactive oxygen species [1]. Oxidative stress caused by cigarette smoking can dysregulate cell function, and induce the damage and death of the cellular constituents of the lungs [2]. Smoking is a major cause of complex lung diseases such as chronic obstructive pulmonary disease. It is important to investigate its effect in “normal” lung at the molecular level to gain a better understanding before investigating its effect in complex lung diseases. Several studies have shown smoking-induced changes in gene expression patterns in the lung. Epithelial cells are the first cells that encounter the inhaled smoke. Consequently, aberrant gene expression signatures were reported in epithelial cells when comparing current smokers with never-smokers [3,4]. Gene expression can be regulated by microRNAs (miRNAs). These small non-coding RNAs influence their target gene and/or protein expressions by binding based on sequence homology [5]. Marked changes in miRNA expression signatures have also been reported in the bronchial airway epithelial cells, small airway epithelium, whole blood, and induced sputum supernatant of current smokers compared to never-smokers [6,7,8,9]. The altered expression signatures may be due to a direct effect of smoking, but can also be caused by indirect effects such as smoking-induced aberrant DNA methylation patterns [10]. Most of the observed changes in gene expression and methylation are (slowly) reversible, while some of the effects may be permanent [4,10]. A previous study showed that when smokers quit smoking for three months, 65% of the miRNAs that were differentially expressed between current smokers and never-smokers return to the expression level of never-smokers [7].

Lung fibroblasts are the main guardians of connective tissue homeostasis. Therefore, lung fibroblasts, in close concert with other structural cells such as the epithelium, are considered crucial cells for tissue repair and remodeling of the lungs. Cigarette smoke suppresses lung repair by affecting multiple lung cells, as nicely reviewed by Rennard et al. [11]. Specific work in human lung fibroblasts showed that the fibronectin and elastin production was inhibited upon cigarette smoke extract (CSE) exposure [12,13]. In addition, lung fibroblasts were hampered in their proliferation, contractile function, and migration toward fibronectin upon CSE exposure [12,13,14]. Furthermore, human lung fibroblasts showed characteristics of senescence when treated with CSE [15]. 

To date, no information on differential miRNA expression in lung fibroblasts from donors with different smoking statuses is available. We hypothesized that the miRNA expression profile in lung fibroblasts is different in current smoking compared to ex-smoking donors, and that these changes in miRNA expression may affect the function of the fibroblasts. The aim of our study was to identify smoking status-related miRNA expression changes in lung fibroblasts and to assess miRNA-related functions that may be affected by current smoking.

## 2. Results

### 2.1. Subject Characteristics

Clinical characteristics of lung fibroblast, lung tissue, and bronchial biopsy donors are shown in Table 1. No significant difference was observed between current smokers, ex-smokers, and never-smokers in age and forced expiratory volume in one second/forced vital capacity ratio (FEV_1_/FVC). Moreover, the number of pack-years did not differ between current smokers and ex-smokers. Furthermore, the characteristics of subjects from whom we have obtained lung fibroblasts and those from whom we obtained lung tissue were not significantly different.

### 2.2. Differential miRNA Expression in Lung Fibroblasts of Current and Ex-Smokers

**Figure 1:**
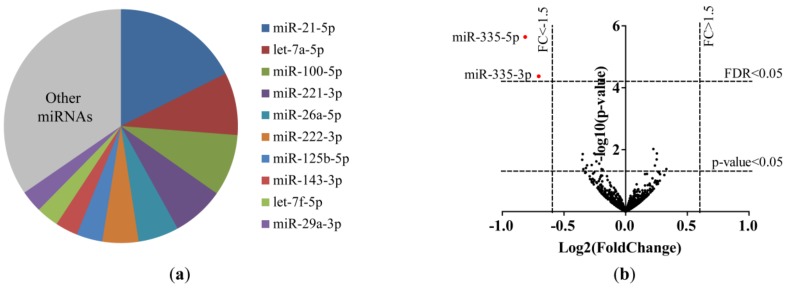
MicroRNAs (miRNAs) in primary lung fibroblasts of ex-smokers and current smokers. (**a**) Top 10 most abundant miRNAs in primary lung fibroblasts of ex-smokers and current smokers. (**b**) Volcano plot of the 1339 miRNAs included in the analyses of the small RNA-sequencing data from lung fibroblasts. The lowest horizontal line represents the nominal *p*-value cut-off of 0.05. The upper horizontal line represents the false discovery rate (FDR) of 0.05. The two vertical lines represent the negative (left) and positive (right) fold change of 1.5. Differentially expressed miRNAs are indicated with a red dot. MiR-335-5p (FC = –1.8, FDR *p*-value = 0.0030) and miR-335-3p (FC = –1.6, FDR *p*-value = 0.0285) were lower expressed in current smokers compared to ex-smokers.

The miRNA expression profiles of lung fibroblasts of nine ex-smoking and six current smoking subjects were determined using small RNA sequencing. Total read counts and percentages of reads mapping to miRBase Release 21 are shown in Table S1. The top 10 most abundant miRNAs in both current smokers and ex-smokers covered 65% of all reads (Figure 1a). MiR-21-5p was the most abundant miRNA in both subgroups.

**Figure 2:**
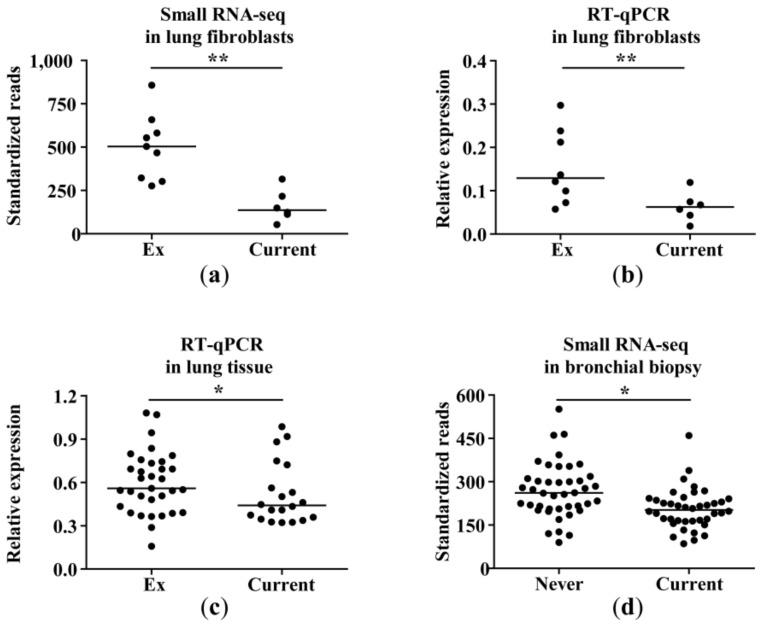
Differentially expressed miR-335-5p in current smokers. (**a**) Standardized reads of miR-335-5p in lung fibroblasts of ex-smokers and current smokers, derived from small RNA sequencing data, as presented in Figure 1. ** FDR *p*-value = 0.0030. (**b**) Validation of miR-335-5p differential expression in the same lung fibroblasts samples using RT-qPCR. The data are presented as relative expression to RNU48 (2^−ΔCp^). One ex-smoker sample is missing due to a failure in experimental procedures. ** *p*-value = 0.0100. (**c**) MiR-335-5p RT-qPCR analysis in lung tissues of ex-smokers and current smokers. The data are presented as relative expression to RNU48 and RNU44 (2^−ΔCp^). * *p*-value = 0.048. (**d**) MiR-335-5p standardized read counts from small RNA sequencing data of bronchial biopsy samples. * *p*-value = 0.018.

A total of 18 miRNAs (five upregulated and 13 downregulated) differed between current smokers and ex-smokers at a nominal *p*-value of <0.05 (Table S2). At a false discovery rate (FDR) cut-off <0.05, miR-335-5p and miR-335-3p were significantly differentially expressed with lower expression levels in current smokers compared to ex-smokers (fold change (FC) = –1.8, FDR *p*-value = 0.003 and FC = –1.6, FDR *p*-value = 0.0285, respectively; Figure 1b, Figure 2a, Figure S1a). Differential expression of miR-335-5p (*p*-value = 0.01, Figure 2b), but not miR-335-3p (Figure S1b), was validated using RT-qPCR in the same samples. 

### 2.3. Validation of miR-335-5p Differential Expression in Lung Tissue and Bronchial Biopsies

We validated the differential miR-335-5p expression in lung tissue from 20 current smokers compared to 33 ex-smokers (*p*-value <0.05, Figure 2c). In lung tissue samples of never-smokers, miR-335-5p expression was not significantly different from current smokers (Figure S2). In bronchial biopsies of healthy subjects with normal lung function, we observed a significantly lower expression of miR-335-5p in current smokers compared to never-smokers (*p*-value <0.05, FC = –1.2, Figure 2d). 

**Figure 3:**
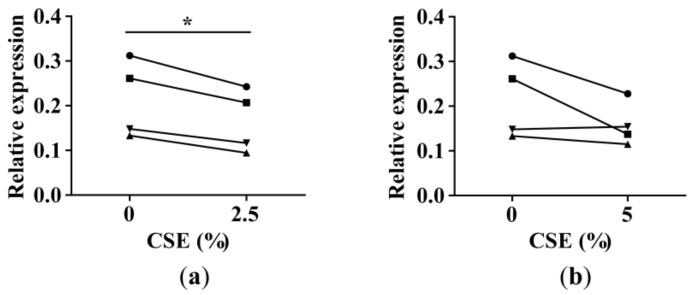
MiR-335-5p expression in cigarette smoke extract (CSE)-treated lung fibroblasts. The CSE treatment started at passage 5 and lasted until the cells had reached a minimum of three cell divisions, i.e., lung fibroblasts were treated with CSE for 21 days. MiR-335-5p expression in lung fibroblasts of four ex-smokers (each individual is indicated with a symbol) treated with (**a**) 2.5% and (**b**) 5% CSE (RT-qPCR). The data are presented as relative expression to RNU48 (2^-ΔCp^). * *p*-value <0.05 using paired T-test.

To assess whether there is a direct smoke effect on miR-335-5p expression, we treated lung fibroblasts from four ex-smokers with 2.5% and 5% CSE. CSE treatment resulted in slightly decreased miR-335-5p levels in all (2.5% CSE) or three out of four fibroblast samples (5% CSE). This experiment supported our findings (Figure 3) pointing toward a CSE-dependent decrease in miR-335-5p levels.

### 2.4. No Regional Hypermethylation in miR-335 Host Gene in Lung Tissue of Current Smokers 

**Figure 4:**
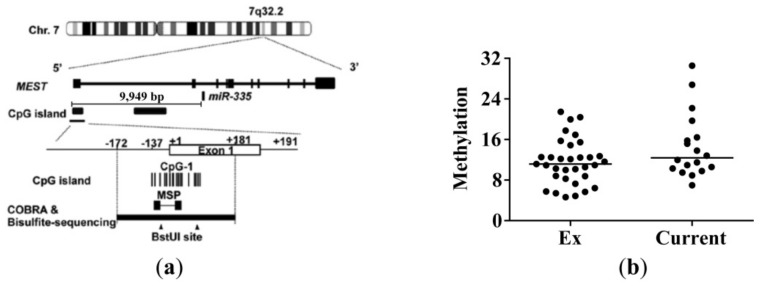
Methylation status in the CpG island of the enhancer region of miR-335-5p in lung tissue. (**a**) Location of the primers for methylation-specific qPCR (MSP) and miR-335 (figure adapted from Dohi et al. [16]). (**b**) Methylation status in specific CpG island was determined in lung tissue from 33 ex-smokers and 18 current smokers. The methylation status was determined as follows: 2^(mean Cp value methylated-specific primers—mean Cp value unmethylated-specific primers). A clear difference was observed between the in vitro methylated and the unmethylated control DNA sample (not shown).

In a previous study of hepatocellular carcinoma, decreased miR-335-5p expression was shown to be associated with the aberrant hypermethylation of a specific CpG island in an enhancer region of the miR-335 host gene [16]. To examine whether the decreased miR-335-5p expression in current smokers is due to hypermethylation, we did a methylation-specific qPCR (MSP) on the lung tissue samples used for measuring the miR-335-5p expression in Figure 2c. The location of miR-335-5p, the analyzed CpG island, and the primers used for the methylation specific qPCR are shown in Figure 4a. We found no differences in the proportion of methylated DNA between current smokers and ex-smokers (Figure 4b). The proportion of methylated DNA was also not correlated with miR-335-5p expression in lung tissue (not shown). Furthermore, the methylation status did not show any obvious change after CSE treatment in lung fibroblasts from ex-smokers (Figure S3a,b). 

### 2.5. Predicted and Experimentally Proven Targets of miR-335-5p in the MiRNA Targetome of Lung Fibroblasts

We previously performed argonaute 2-immunoprecipitation (Ago2-IP) to identify the genes that are actively regulated by miRNAs in primary lung fibroblasts from two control subjects. These genes are collectively called the miRNA targetome [17]. To identify miR-335-5p target genes relevant in lung fibroblasts, we first assessed the enrichment of predicted miR-335-5p target genes in our previously published miRNA targetome of lung fibroblasts. In both controls, we observed 16 predicted miR-335-5p target genes in the top 1500 Ago2-IP enriched probes; however, this was not a significant enrichment compared to the proportion of miR-335-5p targets in all expressed genes (Table S3). 

Next, we identified 40 experimentally proven target genes of miR-335-5p based on published luciferase reporter assays [18,19,20,21,22,23,24,25,26,27,28,29,30,31,32,33,34,35,36,37,38,39,40,41,42,43,44,45,46,47,48,49,50,51,52,53,54,55] (Table S4). Of these genes, *RB transcriptional corepressor 1* (*Rb1*) [18,19], *calcium responsive transcription factor* (*CARF*) [20], and *serum/glucocorticoid regulated kinase family member 3* (*SGK3*) [21] were present in the miRNA targetome of lung fibroblasts. In the RNA sequencing dataset of bronchial biopsies [56], we observed an increase of *SGK3* (FC = 1.1, *p*-value <0.05) in smokers compared to never-smokers, whereas *Rb1* and *CARF* were not significantly different.

## 3. Discussion

In this study, we found miR-335-5p levels to be lower in the parenchymal lung fibroblasts of current smokers compared to those of ex-smokers, and this was validated in lung tissue. Moreover, we also observed a lower miR-335-5p in bronchial biopsies from healthy current smokers compared to never-smokers. A smoking-related decrease in miR-335-5p was supported by decreased miR-335-5p levels upon the CSE treatment of fibroblasts. The lower expression level of this miRNA in the fibroblasts and lung tissue of current smokers was not associated with hypermethylation of the previously reported CpG island. Next, we found that three previously published miR-335-5p target genes, i.e., *Rb1*, *CARF*, and *SGK3*, were present in the miRNA targetome of lung fibroblasts. 

The differential expression of miR-335-5p in lung fibroblasts suggests a potential role of this miRNA in smoking-induced changes in fibroblast function. However, the exact role of miR-335-5p in lung fibroblasts is as yet unknown. In bone-marrow derived human mesenchymal stem cells, the overexpression of miR-335-5p had an inhibitory effect on cell proliferation, migration, and differentiation [34]. This suggests that lower miR-335-5p levels as observed in current smokers and upon CSE exposure in this study could result in enhanced proliferation, as well as in other cell types, such as fibroblasts. However, other studies in lung fibroblasts showed the opposite, i.e., short and long-term CSE exposure reduced the proliferation and migration [13,14]. As the predicted targets of miR-335-5p were not significantly enriched, we searched for the experimentally proven targets. Three of these experimentally proven targets, i.e., *Rb1*, *CARF*, and *SGK3*, were enriched in the Ago2-IP fraction in lung fibroblasts. The presence of these genes in the miRNA targetome shows active targeting by miRNAs, and this might involve targeting by miR-335-5p in lung fibroblasts. As we found a decreased expression of miR-335-5p in lung fibroblast from current smokers, we speculated that these genes might be upregulated in current smokers. *SGK3* is a serine/threonine protein kinase, and to our knowledge, the function of this gene in lung fibroblasts is still unknown. *Rb1* was the most prominently IP-enriched target gene of miR-335-5p. This protein-coding gene can negatively regulate the cell cycle by interacting with E2F Transcription Factor 1 (E2F1), which is required for the activation of genes involved in the S phase of the cell cycle [57]. A previous study showed that nicotine increased Rb1 expression in non-small cell lung cancer cell lines, and the knockdown of Rb1 inhibited cell proliferation [58]. In contrast to this finding, cigarette smoking has been shown to inhibit the proliferation of lung fibroblasts [13]. The second IP-enriched miR-335-5p target was *CARF*, which is a transcriptional activator. CARF was shown to induce the transcription of brain-derived neurotrophic factor (BDNF) exon III in rat neurons [59]. BDNF is also expressed in lung fibroblasts, and it was previously reported that BDNF increased the cell proliferation of lung fibroblasts [60]. However, it is unknown whether CARF also induces BDNF transcription in lung fibroblasts. Additional experiments are required to investigate the role of miR-335-5p and the function of the identified target genes in lung fibroblasts.

Furthermore, miR-335-5p has been reported to be involved in different cancer types, either as a tumor suppressor or as an oncomiR [61]. The downregulation of miR-335-5p in different cancer types was shown to be associated with aberrant DNA methylation [16,62,63]. As lung fibroblasts had differential miRNA expression after isolation and the in vitro culturing of the fibroblasts, it is conceivable that epigenetic changes are involved in the persistent change in miR-335-5p expression. Pilot data using 5-aza-2’-deoxycytidine (not shown) suggested that miR-335-5p expression in fibroblasts indeed may be regulated by DNA methylation. In our study, we focused on a specific CpG island in the miR-335 host gene enhancer region that was reported by Dohi et al. [16]. However, we did not find differences in the methylation status in lung tissue from current smokers and ex-smokers. Moreover, the methylation status of this specific region was also unchanged in lung fibroblasts from ex-smokers after CSE treatment. Thus, our findings suggest that the smoking-related downregulation of miR-335-5p in lung fibroblasts is not due to aberrant DNA methylation at this specific region. However, we cannot exclude that aberrant DNA methylation is present at other regions, which also may affect miR-335-5p expression. In addition to aberrant DNA methylation, cigarette smoke-induced histone modification in the lung has been reported [64], and thus is worthwhile to investigate. 

We showed lower miR-335-5p levels in fibroblasts and lung tissue from current smokers compared to ex-smokers and in bronchial biopsies, as well as compared to never-smokers. However, in lung tissue, we did not observe a difference between current smokers and never-smokers. This could be due to lack of power, as the never-smoking group only consisted of 14 subjects. 

In conclusion, we showed a lower miR-335-5p expression in lung fibroblasts and tissues from current smokers compared to ex-smokers, and in bronchial biopsies from current smokers compared to never-smokers, without a change in the regional methylation pattern of its host gene. The decreased expression of miR-335-5p in lung fibroblasts from current smokers may have an effect on the cell function via targeting *Rb1*, *CARF*, and *SGK3*.

## 4. Materials and Methods

### 4.1. Subjects

Small RNA sequencing was performed on human parenchymal lung fibroblasts isolated from the tissue samples of nine ex-smokers and six current smokers with normal lung function who underwent lung tumour resection surgery. Left-over, macroscopically normal lung tissue samples located far away from the tumor were used for the isolation of lung fibroblasts [65,66]. 

As validation cohorts, we analyzed lung tissue samples of 33 ex-smoking and 20 current smoking individuals with normal lung function by RT-qPCR. These lung tissue samples were also derived from ex-smokers and current smokers with normal lung function who underwent lung tumour resection surgery. In addition, we analyzed data from the bronchial biopsies of 42 never-smoking and of 40 currently smoking healthy individuals with normal lung function and no respiratory symptoms (Clinical Trials Identifier = NCT00848406 [67]). 

This study was performed in accordance with the national ethical and professional guidelines on the use of human body material (“Code of conduct; Dutch federation of biomedical scientific societies”; https://www.federa.org/codes-conduct) and the Research Code of the University Medical Center Groningen (https://www.umcg.nl/SiteCollectionDocuments/English/Researchcode/umcg- research-code-2018-en.pdf). At the time of the experiments, the use of left-over lung tissue to isolate fibroblasts or to replicate the results did not fall within the scope of medical research involving human subjects in the Netherlands. Therefore, an ethics waiver was provided by the Medical Ethical Committee of the University Medical Center Groningen (METc UMCG). All samples and clinical information were de-identified before the start of the experimental procedures in this study.

### 4.2. Isolation, Cell Culture, and CSE Treatment of Primary Lung Fibroblasts

Primary lung fibroblasts were isolated and grown in complete Ham’s F12 medium supplemented with 10% (*v/v*) fetal calf serum (FCS), 100 U/mL penicillin/streptomycin, and 200 mM L-glutamine (all from Lonza, Breda, The Netherlands) and stored in liquid nitrogen until further use as previously described [17,68]. Fibroblast cultures were restored, cultured until passage 5, grown to around 90–100% confluence in complete Ham’s F12 culture medium, and then serum-starved (0.5% (*v/v*) FCS) for 24 hours before harvesting of the cells for RNA isolation.

Fibroblasts of four ex-smoking individuals were treated with 0%, 2.5%, and 5% CSE for 21 days to determine long-term smoke-exposure effects. Two 3R4F research-reference filterless cigarettes (Tobacco Research Institute, University of Kentucky, 12/2006) were bubbled into 25 ml complete Ham’s F12 medium supplemented with 10% (*v/v*) FCS, 100 U/mL penicillin/streptomycin, and 200 mM l-glutamine (all from Lonza) using a peristaltic pump. This was considered as 100% CSE, which was then diluted to 2.5% and 5% CSE in complete medium. The CSE treatment started at passage 5, and lasted until the cells had reached a minimum of three cell divisions. During cell culturing, half of the medium with and without CSE was replaced with fresh medium whenever there was an obvious change in color. The fibroblasts were harvested at passage 7 for RNA and DNA isolation. 

### 4.3. RNA and DNA Isolation

Total RNA was isolated from primary lung fibroblasts and lung tissue samples using TRIzol (Invitrogen, Carlsbad, CA, USA) according to the manufacturer’s protocol. Genomic DNA was isolated using salt–chloroform extraction and isopropanol precipitation using standard procedures. The RNA and DNA concentrations were measured with a NanoDrop 1000 Spectrophotometer (Thermo Scientific, Wilmington, DE, USA). For small RNA sequencing, the RNA quantity and quality were determined using the LabChip GX (Perkin Elmer, Waltham, MA, USA). 

### 4.4. Small RNA Sequencing

Total RNA (approximately 1 μg) was used to generate libraries with the NEXTflex Small RNA-seq kit V3 (Bioo Scientific, Uden, The Netherlands). Sequencing was performed on the NextSeq 500 sequencing system (Illumina, San Diego, CA, USA) according to the protocol of the manufacturer. TrimGAlore 0.3.7 was used to trim the adapter sequences of the raw reads. Subsequently, the reads were allocated to the known human miRNAs allowing one mismatch using the miRDeep2 V2.0.0.8 software [69] and miRBase Release 21 (http://www.mirbase.org/). The reads of miRNAs with the same mature sequence were summed up. Using the default filtering setting of the DESeq2 package in R, miRNAs not expressed in all samples were removed. This resulted in 1339 miRNAs for further analyses. The small RNA sequencing dataset generated for this manuscript is available for collaboration upon request. 

### 4.5. RT-qPCR 

To validate the differential miRNA expression, RT-qPCR was performed as described previously [17]. First, 10 ng of total RNA was reverse-transcribed using a multiplex approach with TaqMan primers (reference gene: RNU48 (Assay ID: 001006) or RNU44 (Assay ID: 001094), ssc-miR-335-5p (Assay ID: 244560_mat) and hsa-miR-335* (hsa-miR-335-3p, Assay ID:002185); Applied Biosystems, Carlsbad, CA, USA) [70]. Subsequently, qPCR was done using TaqMan microRNA assays (Applied Biosystems) and a LightCycler®480 Probes Master (Roche Diagnostics GmbH, Mannheim, Germany). 

The reactions were run in triplicate on the LightCycler®480 Real-Time PCR system (Roche Diagnostics GmbH). The LightCycler®480 software release 1.5.0 (Roche Diagnostics GmbH) was used to analyze the data. The relative miRNA expression levels were calculated using the formula 2^−ΔCp^.

### 4.6. Bisulfite Treatment and Methylation-Specific qPCR

DNA from primary lung fibroblasts and lung tissue samples was treated with bisulfite using the EZ DNA Methylation-Gold™ Kit (Zymo Research, Irvine, CA, USA) according to the protocol of the manufacturer. The DNA of leukocytes in vitro methylated by *Sss*I methyltransferase was used as a positive control, and untreated DNA was used as a negative control [71]. MSP for the miR-335 host gene was done on 10 ng of bisulfite-treated DNA using SYBR green PCR master mix (Applied Biosystems) and previously published methylated-specific primers (forward 5’-TTGTAATAGGTGGCGTTGAC-3’ and reverse 5’-ACTCGAAACTAAAACGTCGC-3’) and unmethylated-specific primers (forward 5’-TTTTTGTAATAGGTGGTGTTGAT-3’ and reverse 5’-ACTCAAAACTAAAACATCACCAA-3’) [16]. For each sample, qPCR with the methylated-specific and unmethylated-specific primers (annealing temperature 58 °C for 1.20 min) were run in triplicate on the same plate. The methylation status was determined as follows: 2^ (mean Cp value methylated-specific primers—mean Cp value unmethylated-specific primers).

### 4.7. Identification of miR-335-5p Targets Relevant for Lung Fibroblasts

We re-analyzed our previously published Ago2-IP data of primary lung fibroblasts from two control subjects to identify miR-335-5p target genes that are Ago2-IP-enriched, and thus targeted by miRNAs in lung fibroblasts [17]. This was done for a list of predicted targets of miR-335-5p identified using TargetScan version 7.2 [72] and for a list of experimentally proven, direct targets of miR-335-5p that was generated based on validation with luciferase reporter assays.

### 4.8. Statistical Analyses

To compare the subject characteristics between and within the study groups, Mann–Whitney U-test was used in IBM SPSS Statistics 20 software. Differential expression analysis of the small RNA sequencing data comparing miRNA expression in the lung fibroblasts of ex-smokers and current smokers, and in bronchial biopsies of never-smokers and current smokers, was performed using the Bioconductor-DESeq2 package (version 1.14.1) in R Project software (version 3.3.2). The data were adjusted for age, gender, and library preparation batch. A Benjamini–Hochberg FDR <0.05 was considered statistically significant. 

For RT-qPCR data, significant differences for miR-335-5p levels in lung fibroblasts and lung tissues between current smokers and ex-smokers were tested using the one-tailed Mann–Whitney U-test. A paired T-test was used to test for miR-335-5p expression changes in CSE-treated lung fibroblasts. A chi-square test was performed on the percentage of predicted targets in the top 1500 enriched probes compared to the percentage of predicted targets in all expressed genes to assess the enrichment of predicted target genes in the Ago2-IP. A *p*-value below 0.05 was considered statistically significant. 

Our previously published RNA-seq data in bronchial biopsies [56] were used to validate the proposed target genes. Differential expression analysis comparing current smokers with never-smokers was performed using the Bioconductor-DESeq2 package (version 1.14.1) in R Project software (version 3.3.2). The data were adjusted for age, gender, and batch. 

## Figures and Tables

**Table 1:** Patient characteristics of the donors of lung fibroblasts, lung tissue, and bronchial biopsy.

Characteristics	Lung Fibroblast Donors		Lung Tissue Donors			Bronchial Biopsy Donors	
	Ex-Smokers	Current Smokers	Never-Smokers	Ex-Smokers	Current Smokers	Never-Smokers	Current Smokers
N	9	6	14	33	20	42	40
Male/Female, *n*	6/3	1/5	6/8	21/12	7/13	23/19	23/17
Age, years ^1^	65.0 (55.0–68.0)	56.5 (48.5–69.0)	56.0 (48.8–73.8)	65.0 (54.0–71.5)	61.0 (51.3–67.8)	38.1 (21.6–57.8)	43.0 (23.4–52.4)
Pack-years, n ^1^	31.5 (17.9–43.1) (*n* = 6)	36.5 (27.8–52.0) (*n* = 6)	NA	33.5 (20.0–46.3) (*n* = 26)	34.0 (20.3–50.8) (*n* = 16)	NA	15.9 (3.9–30.3) (*n* = 40)
FEV_1_, % pred ^1,2^	96.9 (86.8–97.7)	92.4 ^3^	102.0 (91.2–116.5)	90.9 (84.2–104.3)	94.2 (86.1–107.7)	101.2 (92.0–108.6)	97.7 (93.3–107.3)
FEV_1_/FVC, % ^1,4^	76.0 (71.4–79.9)	73.8 (73.1–79.2)	78.0 (72.8–83.0)	73.3 (70.0–78.9)	75.7 (72.6–79.2)	79.5 (75.0–85.4)	78.0 (73.9–83.0)

^1^ Median (interquartile range); ^2^ FEV_1_, % pred = percentage of forced expiratory volume in one second of the predicted normal value for an individual of the same sex, age, and height. ^3^ FEV_1_, % predicted was only available for three out of six current smokers who donated lung fibroblasts. Of these three donors, the FEV_1_ in liters is known. ^4^ FEV_1_/FVC, % = forced expiratory volume in one second/forced vital capacity ratio expressed in percentage. NA = not applicable.

## Supplementary Materials

The Supplementary materials can be found at https://www.mdpi.com/1422-0067/20/20/5176/s1.

## Abbreviations

miRNA	microRNA
CSE	cigarette smoke extract
FEV_1_	forced expiratory volume in one second
FVC	forced vital capacity
FC	fold change
FDR	false discovery rate
MSP	methylation specific qPCR
Ago2-IP	argonaute 2-immunoprecipitation
