# Supplementary material for: Current Smoking is Associated with Decreased Expression of miR-335-5p in Parenchymal Lung Fibroblasts
Source: Int J Mol Sci. 2019 Oct 18;20(20):5176. doi: 10.3390/ijms20205176 (PMC6829537; doi:10.3390/ijms20205176)
Supplement: Supplementary file 1 [file ijms-20-05176-s001.zip › Figure S1_proofreading.docx]

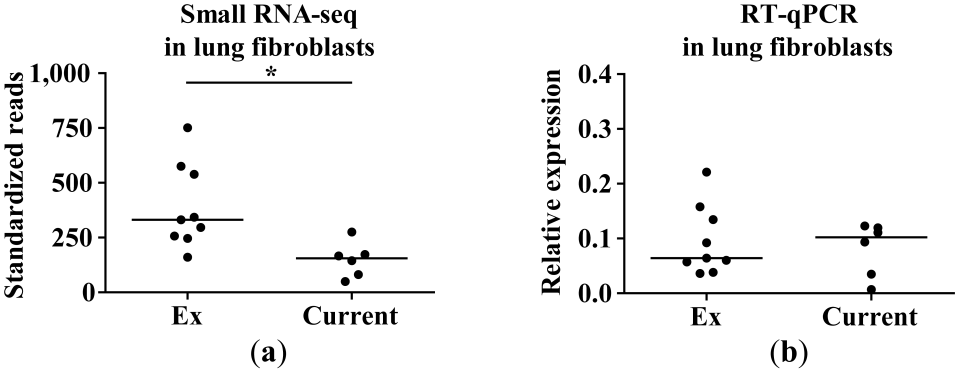


**Figure S1.** Differentially expressed miR-335-3p in current smokers compared to ex-smokers. (**a**) The standardized reads of miR-335-3p in the lung fibroblasts of the ex-smokers and current smokers, derived from the small RNA sequencing data. * FDR *p*-value = 0.0285. (**b**) Validation of miR-335-3p differential expression in the same lung fibroblasts samples using RT-qPCR. The data are presented as relative expression to RNU48 (2^-ΔCp^).
