# Supplementary material for: Current Smoking is Associated with Decreased Expression of miR-335-5p in Parenchymal Lung Fibroblasts
Source: Int J Mol Sci. 2019 Oct 18;20(20):5176. doi: 10.3390/ijms20205176 (PMC6829537; doi:10.3390/ijms20205176)
Supplement: Supplementary file 1 [file ijms-20-05176-s001.zip › Figure S2_proofreading.docx]

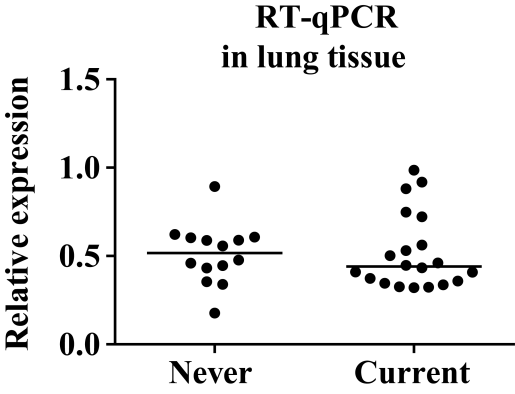


**Figure S2.** MiR-335-5p expression in lung tissue from never-smokers and current smokers. The RT-qPCR miR-335-5p expression data are presented as relative expression to RNU48 and RNU44 (2^-ΔCp^).
