# Supplementary material for: Current Smoking is Associated with Decreased Expression of miR-335-5p in Parenchymal Lung Fibroblasts
Source: Int J Mol Sci. 2019 Oct 18;20(20):5176. doi: 10.3390/ijms20205176 (PMC6829537; doi:10.3390/ijms20205176)
Supplement: Supplementary file 1 [file ijms-20-05176-s001.zip › Figure S3_proofreading.docx]

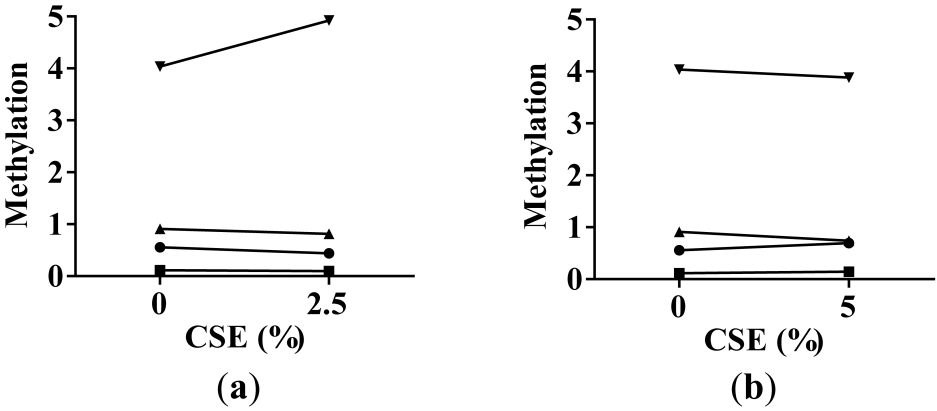


**Figure S3.** Regional methylation status in CSE-treated lung fibroblasts. The methylation pattern in a specific CpG island in the enhancer region of miR-335 host gene [[16](#_ENREF_12)] was determined in the same lung fibroblasts that were treated with (**a**) 2.5% and (**b**) 5% CSE. The methylation pattern was determined as follows: 2^(mean Cp value methylated-specific primers−mean Cp value unmethylated-specific primers).
