# Supplementary material for: Current Smoking is Associated with Decreased Expression of miR-335-5p in Parenchymal Lung Fibroblasts
Source: Int J Mol Sci. 2019 Oct 18;20(20):5176. doi: 10.3390/ijms20205176 (PMC6829537; doi:10.3390/ijms20205176)
Supplement: Supplementary file 1 [file ijms-20-05176-s001.zip › Table S1_proofreading.docx]

**Table S1.** Number of reads obtained by small RNA sequencing and percentage mapped to miRBase.

|  | **Total after trimming** | | **Mapping to miRBase release 21** | |
| --- | --- | --- | --- | --- |
| **Group** | **Sample** | **Reads** | **Reads** | **Percentage** |
| Current smokers | Control 1 | 8,613,228 | 954,721 | 11.1% |
|  | Control 3 | 13,582,970 | 1,310,675 | 9.6% |
|  | Control 4 | 13,664,253 | 1,194,114 | 8.7% |
|  | Control 7 | 15,633,258 | 1,583,379 | 10.1% |
|  | Control 12 | 7,789,705 | 1,213,615 | 15.6% |
|  | Control 15 | 9,459,738 | 1,080,041 | 11.4% |
| Ex-smokers | Control 2 | 5,967,036 | 1,764,687 | 29.6% |
|  | Control 5 | 13,176,488 | 2,280,947 | 17.3% |
|  | Control 6 | 5,447,263 | 1,256,637 | 23.1% |
|  | Control 8 | 7,078,531 | 2,219,801 | 31.4% |
|  | Control 9 | 5,551,348 | 2,396,604 | 43.2% |
|  | Control 10 | 8,892,472 | 1,461,870 | 16.4% |
|  | Control 11 | 16,834,054 | 1,048,153 | 6.2% |
|  | Control 13 | 12,844,518 | 1,314,038 | 10.2% |
|  | Control 14 | 11,511,590 | 1,999,201 | 17.4% |
