# Supplementary material for: Current Smoking is Associated with Decreased Expression of miR-335-5p in Parenchymal Lung Fibroblasts
Source: Int J Mol Sci. 2019 Oct 18;20(20):5176. doi: 10.3390/ijms20205176 (PMC6829537; doi:10.3390/ijms20205176)
Supplement: Supplementary file 1 [file ijms-20-05176-s001.zip › Table S2_proofreading.docx]

**Table S2.** Differentially expressed miRNAs between current smokers and ex-smokers (nominal *p*-value <0.05).

| **miRNA** | **FC** | ***p*-value** | **FDR** |
| --- | --- | --- | --- |
| miR-335-5p | -1.8 | 2.28E-06 | 3.05E-03 |
| miR-335-3p | -1.6 | 4.25E-05 | 2.85E-02 |
| miR-4485-5p | 1.2 | 9.49E-03 | 1.00E+00 |
| miR-1246 | -1.2 | 1.32E-02 | 1.00E+00 |
| miR-3622a-3p | 1.2 | 1.32E-02 | 1.00E+00 |
| miR-195-5p | -1.3 | 1.38E-02 | 1.00E+00 |
| miR-3195 | 1.2 | 2.07E-02 | 1.00E+00 |
| miR-95-3p | -1.3 | 2.11E-02 | 1.00E+00 |
| miR-3661 | -1.2 | 2.39E-02 | 1.00E+00 |
| miR-200c-3p | -1.2 | 2.83E-02 | 1.00E+00 |
| miR-139-5p | -1.2 | 3.13E-02 | 1.00E+00 |
| miR-339-5p | -1.2 | 3.20E-02 | 1.00E+00 |
| miR-5701 | 1.2 | 3.25E-02 | 1.00E+00 |
| miR-146a-5p | -1.3 | 3.89E-02 | 1.00E+00 |
| miR-548h-3p/miR-548z | -1.1 | 4.21E-02 | 1.00E+00 |
| miR-4326 | 1.3 | 4.23E-02 | 1.00E+00 |
| miR-1306-5p | -1.3 | 4.26E-02 | 1.00E+00 |
| miR-486-3p | -1.1 | 4.38E-02 | 1.00E+00 |
