# Supplementary material for: Current Smoking is Associated with Decreased Expression of miR-335-5p in Parenchymal Lung Fibroblasts
Source: Int J Mol Sci. 2019 Oct 18;20(20):5176. doi: 10.3390/ijms20205176 (PMC6829537; doi:10.3390/ijms20205176)
Supplement: Supplementary file 1 [file ijms-20-05176-s001.zip › Table S3_proofreading.docx]

**Table S3.** Predicted miR-335-5p target genes in miRNA targetome of lung fibroblasts.

| **Predicted target genes** | | **Biological processes/pathways** | **Control 1** | **Control 2** |
| --- | --- | --- | --- | --- |
| HIC ZBTB Transcriptional Repressor 2 | *HIC2* | negative regulation of transcription by RNA polymerase II; negative regulation of transcription, DNA-templated | 1 | 1 |
| MON2 Homolog, Regulator Of Endosome-To-Golgi Trafficking | *MON2* | golgi to endosome transport; protein transport | 2 | 7 |
| CTD Small Phosphatase Like 2 | *CTDSPL2* | protein dephosphorylation; negative regulation of BMP signalling pathway; positive regulation of protein export from nucleus | 3 | 5 |
| Kelch Like Family Member 28 | *KLHL28* | - | 4 | 3 |
| Eukaryotic Translation Initiation Factor 4E Binding Protein 2 | *EIF4EBP2* | TGF-beta pathway; beta-adrenergic signalling; regulation of translation | 5 | 4 |
| Nucleic Acid Binding Protein 1 | *NABP1* | gene expression; DNA repair; mitotic cell cycle checkpoint | 6 | 6 |
| SECIS Binding Protein 2 Like | *SECISBP2L* | selenocysteine incorporation | 7 | 9 |
| Microfibril Associated Protein 3 | *MFAP3* | elastic fibre formation; degradation of the extracellular matrix | 8 | 8 |
| Zinc Finger And BTB Domain Containing 10 | *ZBTB10* | - | 9 | - |
| Cysteine Rich Transmembrane BMP Regulator 1 | *CRIM1* | insulin-like growth factor receptor signalling pathway; negative regulation of endopeptidase and catalytic activity | 10 | - |
| Regulator Of Chromosome Condensation 2 | *RCC2* | cell cycle; cytoskeletal signalling; regulation of fibroblast migration | 11 | 16 |
| Cyclin F | *CCNF* | class I MHC mediated antigen processing and presentation; DNA damage; mitotic cell cycle | 12 | - |
| Kelch Domain Containing 10 | *KLHDC10* | positive regulation of stress-activated MAPK cascade | 13 | - |
| Ligand Dependent Nuclear Receptor Corepressor | *LCOR* | regulation of transcription by RNA polymerase II | 14 | - |
| RNA Binding Fox-1 Homolog 2 | *RBFOX2* | signalling by FGFR2; regulation of alternative mRNA splicing, via spliceosome; mRNA processing | 15 | 11 |
| MYC Associated Factor X | *MAX* | CDK-mediated phosphorylation and removal of Cdc6; MAPK signalling pathway; cell cycle; transcription by RNA polymerase II | 16 | - |
| Spermatogenesis Associated 2 | *SPATA2* | necroptosis; regulation of tumour necrosis factor-mediated signalling pathway; programmed cell death; regulation of inflammatory response | - | 2 |
| Activin A Receptor Type 1C | *ACVR1C* | signalling by NODAL; TGF-beta signalling pathway; apoptotic process | - | 10 |
| Family With Sequence Similarity 107 Member B | *FAM107B* | - | - | 12 |
| DNA Damage Inducible 1 Homolog 2 | *DDI2* | proteolysis; proteasomal protein catabolic process; regulation of protein stability | - | 13 |
| Rho GTPase Activating Protein 18 | *ARHGAP18* | p75 NTR receptor-mediated signalling; signalling by GPCR; regulation of cell shape | - | 14 |
| Ring Finger Protein 141 | *RNF141* | regulation of transcription, DNA-templated; protein autoubiquitination | - | 15 |

MiR-335-5p is broadly conserved and had in total 289 predicted targets. The numbers in the table indicate the rankings of the predicted target genes based on the IP-enrichment, i.e. 1=most IP-enriched predicted target gene, 2=second most IP-enriched target gene.
