# Supplementary material for: Current Smoking is Associated with Decreased Expression of miR-335-5p in Parenchymal Lung Fibroblasts
Source: Int J Mol Sci. 2019 Oct 18;20(20):5176. doi: 10.3390/ijms20205176 (PMC6829537; doi:10.3390/ijms20205176)
Supplement: Supplementary file 1 [file ijms-20-05176-s001.zip › Table S4_proofreading.docx]

**Table S4.** Reported proven miR-335-5p target genes in miRNA targetome of lung fibroblasts.

| **Enriched in miRNA targetome**  **of lung fibroblasts** | **Proven targets** | | **Biological processes/pathways** | **Refs** | |
| --- | --- | --- | --- | --- | --- |
| Yes | RB Transcriptional Corepressor 1 | Rb1 | cyclins and cell cycle regulation; G1/S transition of mitotic cell cycle; regulation of cell growth | [[18](#_ENREF_18),[19](#_ENREF_19)] | |
|  | Calcium Responsive Transcription Factor | CARF | regulation of transcription, DNA-templated; cellular response to potassium ion; cellular response to calcium ion | [[20](#_ENREF_20)] | |
|  | Serum/Glucocorticoid Regulated Kinase Family Member 3 | SGK3 | ion channel transport; PI3K / Akt signalling; regulation of cell growth; regulation of cell migration | [[21](#_ENREF_21)] | |
| No | Poly(ADP-Ribose) Polymerase 1 | PARP1 | telomere C-strand (Lagging Strand) synthesis; DNA double-strand break repair; negative regulation of transcription by RNA polymerase II | [[22](#_ENREF_22)] | |
|  | Sp1 Transcription Factor | Sp1 | IL-2 pathway; transcriptional activity of SMAD2/SMAD3-SMAD4 heterotrimer; regulation of transcription, DNA-templated | [[23](#_ENREF_23),[25](#_ENREF_25),[26](#_ENREF_26)] | |
|  | Dishevelled Associated Activator Of Morphogenesis 1 | DAAM1 | Wnt signalling pathways: beta-Catenin-dependent Wnt signalling; cellular component organization; actin cytoskeleton organization | [[27](#_ENREF_27)] | |
|  | RAS P21 Protein Activator 1 | RASA1 | RET signalling; MAPK signalling pathway; negative regulation of cell-matrix adhesion | [[28](#_ENREF_28)] | |
|  | EPH Receptor A4 | EPHA4 | GPCR pathway; ERK signalling; cell adhesion | [[29](#_ENREF_29)] | |
|  | Taurine Up-Regulated 1 | TUG1 | regulation of eye photoreceptor cell development | [[30](#_ENREF_30)] | |
|  | Formin 2 | FMN2 | cellular response to DNA damage stimulus; protein transport; meiotic chromosome movement towards spindle pole | [[31](#_ENREF_31)] | |
|  | Insulin Like Growth Factor 1 Receptor | IGF1R | apoptotic pathways in synovial fibroblasts; protein phosphorylation; immune response; positive regulation of cell proliferation | [[23](#_ENREF_23)] | |
|  | Dishevelled Associated Activator Of Morphogenesis 2 | DAAM2 | Wnt signalling pathways: beta-Catenin-dependent Wnt signalling; determination of left/right symmetry; cellular component organization | [[31](#_ENREF_31)] | |
|  | Copine 1 | CPNE1 | innate immune system; glycerophospholipid biosynthesis; proteolysis; cell differentiation | [[32](#_ENREF_32)] | |
|  | Mitogen-Activated Protein Kinase 1 | MAPK1 | RET signalling; IL-2 pathway; MAPK cascade; apoptotic process | [[33](#_ENREF_33)] | |
|  | SRY-Box 4 | SOX4 | signalling by Wnt; microRNAs in cancer; skeletal system development | [[34-36](#_ENREF_34)] | |
|  | Intercellular Adhesion Molecule 1 | ICAM1 | blood-brain barrier and immune cell transmigration: VCAM-1/CD106 signalling pathways; ovarian follicle development; interferon gamma signalling | [[37](#_ENREF_37)] | |
|  | Zinc Finger E-Box Binding Homeobox 2 | ZEB2 | microRNAs in cancer; TGF-beta receptor signalling; negative regulation of transcription by RNA polymerase II | [[38](#_ENREF_38)] | |
|  | Estrogen Receptor 1 | ESR1 | prolactin signalling pathway; gene expression; chromatin remodelling | [[23](#_ENREF_23)] | |
| **Enriched in miRNA targetome**  **of lung fibroblasts** | **Proven targets** | | **Biological processes/pathways** | | **Refs** |
| No | Cadherin 11 | CDH11 | cell junction organization; ERK signalling; cell morphogenesis; cell adhesion | | [[39](#_ENREF_39)] |
|  | RUNX Family Transcription Factor 2 | RUNX2 | gene expression; Notch signalling pathway; skeletal system development | | [[34](#_ENREF_34)] |
|  | MET Proto-Oncogene, Receptor Tyrosine Kinase | MET | RET signalling; apoptotic pathways in synovial fibroblasts | | [[40](#_ENREF_40)] |
|  | Plasminogen Activator, Urokinase Receptor | PLAUR | innate immune system; metabolism of proteins; chemotaxis | | [[41](#_ENREF_41),[42](#_ENREF_42)] |
|  | BCL2 Like 2 | BCL2L2 | apoptosis modulation and signalling; apoptosis and autophagy; direct p53 effectors | | [[25](#_ENREF_25),[26](#_ENREF_26),[43](#_ENREF_43),[44](#_ENREF_44)] |
|  | Tenascin C | TNC | integrin pathway; ERK signalling; cell adhesion; positive regulation of cell proliferation | | [[35](#_ENREF_35)] |
|  | Lysine Demethylase 4C | KDM4C | activated PKN1 stimulates transcription of AR (androgen receptor) regulated genes KLK2 and KLK3; chromatin organization; blastocyst formation | | [[45](#_ENREF_45)] |
|  | Paired Box 6 | PAX6 | regulation of beta-cell development; incretin synthesis, secretion, and inactivation; cell fate determination | | [[46](#_ENREF_46)] |
|  | Twist Family BHLH Transcription Factor 1 | TWIST1 | cytokine signalling in immune system; interleukin-4 and 13 signalling; negative regulation of transcription by RNA polymerase II | | [[47](#_ENREF_47)] |
|  | Formin Like 3 | FMNL3 | signalling by Rho GTPases; angiogenesis; regulation of cell shape | | [[31](#_ENREF_31)] |
|  | Protein Tyrosine Phosphatase Receptor Type N2 | PTPRN2 | innate immune system; PAK pathway; protein dephosphorylation | | [[35](#_ENREF_35)] |
|  | Baculoviral IAP Repeat Containing 5 | BIRC5 | apoptosis modulation and signalling; cell cycle, mitotic; protein phosphorylation | | [[48](#_ENREF_48)] |
|  | TNF Superfamily Member 11 | TNFSF11 | PEDF induced signalling; ossification; immune response | | [[49](#_ENREF_49)] |
|  | POU Class 5 Homeobox 1 | POU5F1 | human early embryo development; Wnt / Hedgehog / Notch; negative regulation of transcription by RNA polymerase II | | [[50](#_ENREF_50)] |
|  | Rho Associated Coiled-Coil Containing Protein Kinase 1 | ROCK1 | blood-brain barrier and immune cell transmigration: VCAM-1/CD106 signalling pathways; development Slit-Robo signalling; mitotic cytokinesis | | [[24](#_ENREF_24),[30](#_ENREF_30),[33](#_ENREF_33),[51](#_ENREF_51)] |
|  | WW Domain Binding Protein 5 | WBP5 | - | | [[52](#_ENREF_52)] |
|  | RB Binding Protein 8, Endonuclease | RBBP8 | DNA double-strand break repair; cell cycle checkpoints; DNA replication | | [[53](#_ENREF_53)] |
|  | Transformer 2 Beta Homolog | TRA2B | mRNA splicing - major pathway; gene expression | | [[54](#_ENREF_54)] |
|  | C-X-C Motif Chemokine Receptor 4 | CXCR4 | Akt signalling; EphB-EphrinB signalling; activation of MAPK activity | | [[41](#_ENREF_41)] |
|  | Leucine Rich Alpha-2-Glycoprotein 1 | LRG1 | innate immune system; positive regulation of endothelial cell proliferation; positive regulation of transforming growth factor beta receptor signalling pathway | | [[33](#_ENREF_33)] |
|  | MER Proto-Oncogene, Tyrosine Kinase | MERTK | GPCR pathway; natural killer cell differentiation; phagocytosis | | [[35](#_ENREF_35)] |
|  | Tripartite Motif Containing 29 | TRIM29 | interferon gamma signalling; DNA damage; immune system process | | [[55](#_ENREF_55)] |
